# Congress of Gynæcology and Obstetrics

**Published:** 1899-03

**Authors:** 


### Congress of Gynæcology and Obstetrics.

The Third Session of the International Congress of Gynæcology and Obstetrics will be held at Amsterdam, from the 8th to the 12th of August, 1899. The questions arranged for discussion are as follows:—1. The surgical treatment of fibro-myoma. 2. The relative value of antisepsis and improved technic for the actual results in Gynæcological Surgery. 3. The influence of posture on the form and dimensions of the pelvis. 4. The indication for Cæsarean section compared to that for symphysiotomy, craniotomy, and premature induction of labour.

